# Climatology and Sanitation

**Published:** 1899-10

**Authors:** T. S. Hopkins

**Affiliations:** Thomasville, Ga.


### CLIMATOLOGY AND SANITATION.

By T. S. HOPKINS, M.D.,
THOMASVILLE, GA.

Our climate of last winter, counting from November 1, 1898, to February 28, 1899, though reported in Northern papers as an unusual and remarkably cold winter, cannot be classed as such with us. The minimum of temperature from November 1 to February 12, was 21°. Many previous winters have given lower minimums than this. On February 12 it rained, hailed, sleeted, and snowed, and during the night the mercury fell to 2°. On the 14th it rose to 12° and continued to rise and soon the cold wave was gone. The cold Monday of February 13, 1899, was the coldest day in the South of which I have any knowledge, and will become as memorable in the future as has been the cold Saturday of 1835 in the past. Meteorologists define climate as that condition of the atmosphere which influences organized beings, the man, the horse, the cow, the rabbit, the gigantic tree, the chicken, the rose, and the little violet, all of which are organized beings. Having experienced the atmospheric condition of the cold Saturday of 1835, and the cold Monday of 1899, I feel authorized to speak and write *ex cathedra* of the influence of the atmosphere on organized beings.

During the cold Saturday of 1835, said to have been the coldest in the South for a hundred years, I was in Athens, Ga., wearing Canada snow boots, chasing rabbits half knee deep in snow. On the cold Monday of 1899, I was in my cozy little room at Thomasville by a blazing fire, and if a poor little rabbit had been seen passing my door, instead of chasing him unto death, I would have invited him to come in and partake of my warmth. I have a very high opinion of the rabbit. In thermometry and meteorology he has been to me an educator. I have treated a great many of them and have always found that the kinder I was to them the more grateful they seemed to be to me. No kindness to the rabbit could induce him to personate the frozen Adder. The cold wave of February, 1835, left no mementoes in the shape of chickens with amputated combs and gills and feet, hobbling about on stumps. The cold wave of February, 1899, left many. These cold waves or anticyclones or low area storms come to us from the barren land of British America, where terrestrial radiation is so rapid as to chill the air which is driven by northerly winds down the valley of the Mississippi to the southwest, following the receding footsteps of a preceding cyclone or a high area storm which has lifted and carried with it the heated air to the northeast. If there was a range of mountains extending transversely from the Rocky Mountains to the Mississippi River, we would have no more cold waves. The Appalachian chain affords us some protection.

During the past winter my observations were mainly confined to the frequency and amount of precipitation. During the months of November, December, January and February, 1898 and 1899, I carried continuously in my coat pocket a note-book and pencil, and whenever there was any precipitation whatever, a sprinkle, a trace, or a downpour, the date of the precipitation was recorded. From this note-book I find that in November the days of precipitation were, 5th, 10th, 11th, 12th, 14th, 15th, 16th, 17th, 18th, 22d, 25th, 29th; number of days 13. December 2d, 3d, 7th, 9th, 10th, 12th, 15th, 16th, 17th, 18th, 19th, 20th, 21st, 22d, 24th, 26th, and 27th; number of days 17. January 5th, 6th, 9th, 10th, 12th, 13th, 14th, 17th, 19th, 23d, 24th, 26th, 27th, and 31st; number of days 14. February 2d, 3d, 5th, 7th, 8th, 10th, 11th,

12th, 13th, 14th, 15th, 16th, 18th, 21st, 22d, 26th, 27th; number of days 18. Total number of days for period 120. Days of precipitation 62. Without precipitation 58.

During the period from which this report is made, my object was to determine the relative and absolute humidity of the atmosphere. The result proves that while our winter was not a very cold winter, there having been but one very cold spell, lasting only 24 hours, yet for frequency of precipitation, relative and absolute humidity and percentage of cloudiness, it has rarely if ever been surpassed. If misery loves company it may be a consolation to us to know that there was not in the entire South a section not subjected to the same atmospheric condition at the same time. Northern newspapers which represent the past winter as unparalleled in the history of cold winters have not recently referred to the records of the past, or have forgotten the winter of 1878, when the mercury was 60 below zero in the northwest, and when in 1883 it was 63, and hundreds of animals were frozen to death, and travelers caught in the blizzard were compelled to kill the animals on which they rode, disembowel them and crawl into the cavity to save their lives. There is nothing new under the sun. There never has been a winter climate without a precedent.

I am indebted to Mr. Robert Thomas, of the Weather Bureau of this place, for the following reports of the temperature and rainfall for November, December, January and February. November, maximum 82, minimum 30, mean 56, rainfall 4.8. December, maximum 74, minimum 26, mean 50, rainfall 5.05. January, maximum 75, minimum 30, mean 51, rainfall 3.75. February, maximum 80, minimum 22, mean 41, rainfall 4.12. Total rainfall for period, 17.72.

By way of comparison I give report of Mr. McSwain for the same months. This report gives the absolute maximum, minimum, mean temperature and rainfall based upon six years of observation. November, maximum 84.5, minimum 26, mean 55, rainfall 2.64. December, maximum 79.5, minimum 10, mean 44, rainfall 3.81. January, maximum 78, minimum 14, mean 41, rainfall 3.47. February, maximum 82, minimum 28, mean 55, rainfall 3.30. Total rainfall 13.16. According to Thomas, the winter mean was 49.2, McSwain 51.2; my own observations for many years were 54.

On comparing these reports with reports before me for spring, summer and autumn, we may approximately fix our annual rainfall according to Thomas at 58 inches, McSwain 54 inches. It is next to impossible to fix accurately the rainfall at any locality. The rainfall at any place depends upon its exposure to the prevailing rain-bearing winds. We are exposed mainly to the winds from the southwest, coming to us from the Gulf of Mexico, where nature's distillery is ever at work lifting aqueous vapor from the salt water into the atmosphere, when it is wafted over us by the prevailing winds, and when reaching a sufficient altitude to reduce it to saturation, falls upon us as rain, furnishing us with our wells and springs from which we drink. We have no reason to complain of our daily, monthly or annual rainfall. The greatest daily rainfall of which I have any knowledge was 5 inches. The greatest monthly was 14.70, and the greatest annual has never exceeded 60 inches.

Now let us compare our monthly and daily rainfalls with other sections in the South. At Brownsville, Texas, September, 1886, it was 30.6; at Fernandina, Fla., 1864, it was 28.9; Merritt's Island, Fla., 1878, it was 28; Opelika, Ala., July, 1887, it was 20.14; Fort Barankas, Fla., in 1878, it was 30.73; Thomasville, Ga., February, 1897, it was 14.70. Daily rainfall, Thomasville, Ga., 5 inches; Mayport, Fla., September 21, 1885, 9.5 inches, September 29, 1882, 13.7 inches; Savannah, Ga., August 6, 1872, 9.6 inches; Pensacola, Fla., June 28, 1889, 10.7. Having compared our annual, monthly and daily rainfalls with other sections, I will compare the hourly rainfalls. Thomasville, Ga., in four hours, 4 inches; Biscayne, Fla., March 28, 1871, in thirty minutes, 4.10 inches; Galveston, Texas, June 4, 1871, in fourteen minutes, 3.99 inches.

It is difficult to explain the cause of these immense rainfalls at different periods in different localities. If all the moisture in the atmosphere above any locality was suddenly reduced to saturation and fell as rain, the amount would not exceed 2 inches. The only explanation is that the attraction of one locality for the moisture of another is the cause. It must not be inferred from these reports of occasional immense rainfalls at different localities that those localities are damp and unhealthy. The humidity of a climate depends much more upon the nature of the soil than the amount of rainfall. A sandy soil through which the water quickly percolates and disappears, would add less humidity to the atmosphere with double the amount of rainfall than a clay soil from which much escapes by evaporation.

I have given in this article the mean temperature for months. There is but little significance in it whether coming from a weather bureau or a health resort. Like charity in religion it covers a multitude of sins, thus: February 13, 1899, the maximum was 82, minimum 2, and mean 41; December 31, 1880, maximum 79, minimum 10, mean 44. In the first there was a monthly range of 78, in the last 69. I have selected these periods because they give the lowest minimums at Thomasville for 19 years. The mean temperature of winter it will be seen is based upon two days of observation. Of intermediate days we have no report. To be honest in our reports of climate we must find and prove our mean temperature for days, months and years by the variability of the mean temperature of days, months and years. To do this we must divide the sum of the difference of the daily means by the number of days of observation. We hear nothing of the variability of the daily mean in reports of climatologists.

Three thousand years ago Hippocrates, the father of medicine, taught that the prerequisites to health were pure air, pure water, and pure soil. If our sanitarians of the present day would be guided by such teaching it would be well for us. If our air is impure, it comes to us from an impure soil. If our well water is impure, the impurity comes to it from the impure soil through which it percolates. Within the last few years artesian water has been credited with great sanitary powers, and there is some truth in it. The artesian water comes to us from the mountains after percolating through hundreds of miles of filtrating soil by which it is defecated. Deep well water, whether from a depth of 500 or 2,000 feet, is liable to be polluted by unsanitary environments. The artesian well in Atlanta, with a depth of 2,000 feet and at a cost of $30,-

000, after a few years of use was condemned and filled up because the water was polluted. This was not a flowing well. We have no such reports from artesian wells—wells that flow. The difference between artesian water, flowing water, and deep well, non-flowing water, is simply this: The first reaches us after passing through hundreds of miles of filtrating soil, and is filtered water, and when the crust of the reservoir which contained it is punctured by the antagonizing powers of pressure and resistance, is forced many feet above the surface without mingling with surface or soil water. Deep well, non-flowing water is surface water continually descending directly to the strata of horizontal rock, in the crevices of which it circulates, and when a well is bored into the rock, flows into it and may rise many feet in the curb, according to the depth of bore through the rock from the crevices of which it receives its supply. This water may, and has been polluted by the unsanitary surroundings. While the sanitary powers of artesian water have been demonstrated in many cities by their improved health condition, I do not believe that *per se*, it will prove prophylactic or antidotal in malarial trouble. During the epidemic of yellow fever in 1893, at Brunswick, ships from all parts of the world entered the harbor, took on cargoes of lumber and sailed away. I am credibly informed that during the epidemic not a case of yellow fever occurred on one of these ships, while within two or three hundred feet of them the epidemic prevailed. The sailor and the citizen used the same artesian water. The sailor was on the water and escaped. The citizen was on the impure soil from which emanated the pathogenic germ of the pestilence. Since Brunswick has been thoroughly sewered and the soil-water drained off by subsoil pipes, there is no healthier city on the coast of Georgia.

Professor Loomis, of Yale College, a distinguished meteorologist, says: "Climate depends upon the mean temperature of the year, upon that of each month and each day; upon the maximum and minimum temperature; upon the frequency and suddenness of atmospheric changes; upon the temperature of the atmosphere; upon the moisture of the air and the earth; upon the prevalence of fogs and dew; upon the amount of rain and snow; the frequency of thunder storms and hail; the direction and dryness of winds. All these particulars can only be determined by long and careful observation." It is amusing to read letters from "tourists" on their return from a sojourn of a few weeks at a health resort in which the outside world is thoroughly posted on the climate.

This report was intended for the winter, but I will say in conclusion something of the summer. Once in the last twenty-five years in the month of July the temperature here reached 101.5; at Savannah, in July, 1879, 105; at Montgomery, Ala., in July, 1881, 106.5; at Fort Smith, Ark., 1886, 104.5; at Jacksonville, Fla., July, 1875, 104; Augusta, Ga., 1878, 106. These reports are not from newspapers, but from officers of the United States Weather Bureau. While we may complain of our long summers, sometimes from May to the middle of October, we have no reason to complain of extreme heat. Our summer nights, fanned by the Gulf wind, are generally cool and pleasant, affording us refreshing sleep, and in the morning we awake renovated from any enervation caused by any heat of the preceding day. What I have said of our immediate section applies to all sections of our State having the same latitude, longitude, topography, and exposure to prevailing winds. We cannot create a climate, nor alter the direction of the winds, nor reduce the amount of rainfall, nor control the temperature of the atmosphere, but we can, by scientific sanitary work, lessen the relative and absolute humidity, purify the soil, prevent impurity of the atmosphere, and diminish largely the percentage of disease and mortality in any locality.

The study of climatology naturally leads the physician to the study of climato-therapy, that, having acquired a knowledge of the remedial adaptability of different climates to the different forms of disease coming under his observation he may utilize them as prescriptions. Climatic treatment, like medical treatment, is tentative, and it is only by long and careful experimentation that we learn the modus operandi and therapeutic action of either. During the last twenty years, many scientific physicians uninfluenced by personal interest or the hope of financial reward, working wholly and solely in the cause of science and humanity, have devoted their time to the investigation of this subject. From these scientific men come reports from all parts of the country on which is based the consensus of medical opinion, that the hope of the asthmatic and consumptive for relief is to be found is high altitudes from five to eight thousand feet above the sea level, where the atmosphere is almost absolutely dry. This opinion is substantiated by the fact that when a consumptive has been cured, or seemingly cured, in high altitudes, on returning to a low altitude where the climate is warm and moist, with very few or any exceptions, he soon relapses and dies.

My first experiment in testing the therapeutic action of high altitudes in the treatment of consumption was made in 1853. During the summer of that year I advised three young friends and patients of mine to go on to the "Western Plains" or "Great American Desert," then inhabited by the buffalo and Indian only. Two of these young men, the sons of a wealthy rice planter, presented signs of incipient phthisis. The other was supposed to be a hopeless case of consumption. They followed my advice and before reaching Pikes Peak, where they pitched their tents, their improvement was phenomenal, and the "hopeless case" was, with his companions, chasing and killing buffalo. After six months of sojourn in the mountains they returned to their piny woods homes in Wayne county, Georgia, looking well, feeling well and believing they were well. Very soon with two of them the cough returned, they relapsed, and in less than three years they filled consumptive graves. The other, feeling that he was not doing well, sold his two hundred negroes and plantation and emigrated to Western Texas, where he is still living and pursuing his legal profession. Had the others followed his example they would probably be living now. The oft-repeated results in such cases substantiates the therapeutic adaptability of high altitude and dry climate over low altitude and damp climate. Prior to the discovery of the tubercle bacillus by Dr. Koch, and his proof by inoculation with the sputa of the consumptive, that consumption was an infectious disease, "heredity" as the cause hung like a pall over every descendant of a consumptive ancestor. Now, the offspring of consumptive parents know that by a strict avoidance of mingling with the consumptive, the occupancy of rooms previously occupied by a consumptive, especially carpeted rooms, a life out of doors and indulgence in healthy nourishing foods, there is no reason why they should not escape the fate of their ancestors.

There is no race or class of people among which the infectious nature of consumption has been more clearly elucidated and demonstrated than the negro. Prior to emancipation a consumptive negro was somewhat of a curiosity. Now, in the same climate where he was born and reared and approximately immune, he is being decimated by it. Contracting it in boarding-houses and hotels, the winter habitats of the consumptive, by his cosmopolitan habits, he has transmitted it to his race throughout the South.

While indorsing and recommending high altitudes for the consumptive, we do not include all cases of that disease. To the consumptive where there is much destruction of lung tissue, with weak heart which generally follows, high altitude would be perilous, if not fatal. If such cases determine to abandon the comforts of home in the futile effort to catch at a straw, low altitudes would be best for them. Chronic bronchitis, laryngitis, weak hearts from any cause, Bright's disease, diabetes and the whole catalogue of "nervous troubles" do best in low altitudes where the climate is warm and moderately damp.

The intelligent white man, educated to know that consumption is an infectious disease, can escape it by avoiding the sources of infection, but for the ignorant negro who prefers a life of aggregation rather than segregation, there is no hope.

In 1782 consumption prevailed to such an alarming extent in Italy, especially in that portion then the kingdom of Naples, that laws were passed to check its spread. The disease was declared a contagious disease and a returnable disease. The penalty for not reporting a case of consumption was a fine of three hundred ducats ($684.00). This was for the first offense, and upon repetition, banishment from the kingdom for ten years. Any layman who assisted in an evasion of the law was sent to prison. The clothes of the infected person were destroyed by fire, infected houses were thoroughly renovated, the walls replastered and the sick sent to hospitals and isolated. As a result of this law rigorously enforced, the death-rate in Italy from consumption was reduced from ten to one and one-half.

In England there has been in the last forty years a reduction of fifty per cent. in the death-rate from consumption by following the example of Naples, where the disease has been nearly exterminated. When these laws were being enforced the microscopic searchlight of Koch had not brought to view the bacillus, the contagium vivum, the living germ, the architect and builder of the tubercle. Koch has taught us that consumption is not only a communicable disease, but a preventable one, and, fortunately for the human race, has taught us how to prevent it.

